# Systematic Review with Meta-Analysis: *Lactobacillus reuteri* DSM 17938 for Treating Acute Gastroenteritis in Children. An Update

**DOI:** 10.3390/nu11112762

**Published:** 2019-11-14

**Authors:** Bernadeta Patro-Gołąb, Hania Szajewska

**Affiliations:** Department of Paediatrics, The Medical University of Warsaw, 02-091 Warsaw, Poland; bernadeta.patro-golab@wum.edu.pl

**Keywords:** systematic review, probiotics, microbiota, diarrhea, infants, children

## Abstract

The effectiveness of *Lactobacillus reuteri* DSM 17938 (*L. reuteri*) for the management of acute gastroenteritis (AGE) has been recently questioned. We performed a systematic review to update evidence on *L. reuteri* for treating AGE in children. We searched MEDLINE, EMBASE, the Cochrane Library databases, and additional data sources from January 2016 (end of search for our 2016 systematic review) to August 2019. The primary outcomes were stool volume and duration of diarrhea. Four RCTs were included. None of them evaluated stool volume. Compared with placebo or no treatment, *L. reuteri* reduced diarrhea duration (four RCTs, *n* = 347, mean difference, MD −0.87 days, 95% CI [−1.43, −0.31]). *L. reuteri* use was also associated with a reduced duration of hospitalization (three RCTs, *n* = 284, MD −0.54 days, 95% CI [−1.09, 0.0]). The small effect sizes of limited clinical relevance and methodological limitations of the included trials should be noted when interpreting these findings.

## 1. Introduction

Acute gastroenteritis (AGE) in children remains a common health problem. Despite treatment advances in recent decades, globally, diarrhea is one of the leading causes of death in the pediatric population, especially in children younger than the age of 5 years [1]. In the developed setting, childhood AGE accounts for significant healthcare system costs, mainly associated with hospital admissions and physician consultations [2]. Although rotavirus vaccination has been introduced recently in many countries, this form of primary prevention has not overthrown the burden of AGE [1]. Consequently, a focus on effective diarrhea management is still timely and of importance.

Probiotics have been extensively studied as a supportive treatment regimen in children with AGE and shown to be effective in reducing both diarrhea duration and the intensity of symptoms [3]. Some evidence also supports their efficacy in reducing the duration of hospitalization [3]. However, as the term ‘probiotics’ refers to a heterogeneous group of live bacteria with species- and strain-specific properties, general statements regarding their effectiveness in the treatment of AGE cannot be applied to each probiotic product. Different *Lactobacillus reuteri* strains have been proposed to act as therapeutic agents in AGE in children. One of the commercially available strains is *L. reuteri* DSM 17938. This strain is derived from *L. reuteri* ATCC 55730 by the removal of two plasmids, resulting in its loss of resistances to tetracycline and lincomycin [4]. The mechanisms explaining beneficial effects of *L. reuteri* DSM 17938 are not clear. However, it has been postulated that these mechanisms may include interference with pathogen attachment, interaction with the gut microbiota, and immunomodulatory effects [5].

In 2016, we performed a systematic review to investigate the effectiveness of *L. reuteri* DSM 17938 in the treatment and prevention of acute diarrhea in children, showing that its intake reduces diarrhea duration and increases the chance of cure [6]. These conclusions were based on results from three randomized control trials (RCTs) [7,8,9] with considerable methodological limitations, that investigated effects of *L. reuteri* DSM 17938 on treatment outcomes. Since then, new contradictory evidence on probiotics has been recently published. For example, evidence from 2 RCTs performed in children with AGE in the US [10] and Canada [11] found no effect of either *Lactobacillus rhamnosus* GG or a probiotic product containing a combination of *L. rhamnosus* R0011 and *L. helveticus* R0052 on outcomes. Of significance to the current update, our recent RCT showed that *L. reuteri* DSM 17938 compared with placebo, as an adjunct to rehydration therapy, reduced the duration of hospitalization but not the duration of diarrhea in children below the age of 5 years with AGE [12]. Given these recent findings, here, we aim to systematically update evidence on the effectiveness of *L. reuteri* DSM 17938 in the treatment of AGE in children. This systematic review was initiated as part of the update of the guidelines for the use of probiotics in the management of AGE in children [13].

## 2. Material and Methods 

The methodology of this review was similar to that applied in our previous systematic review on the same research question (https://doi.org/10.1111/apt.13590) [6]. Thereby, we did not register our protocol. We followed the Cochrane Collaboration guidelines for undertaking this review, and the PRISMA statement with respect to reporting of our study [14]. No ethical approval was needed to perform this systematic review.

### 2.1. Inclusion Criteria for This Review 

All relevant RCTs that compared the administration of *L. reuteri* DSM 17938 (as a single ingredient, regardless delivery vehicles and formulations, at any dose) with no *L. reuteri* DSM 17938 (defined as placebo or no treatment) were eligible for inclusion. The *primary* outcome measures were diarrhea duration and stool volume. The *secondary* outcome measures were the effects of *L. reuteri* DSM 17938 on the course of diarrhea, including the percentages of children with diarrhea at various times intervals (as specified by the investigators), the percentage of children with diarrhea lasting longer than 7 days, the duration of hospitalization in inpatients, and adverse effects. We also considered other outcomes evaluated by the authors of the original trials, if relevant to this review.

### 2.2. Search Methods for Identification of Studies 

We searched the Cochrane Central Register of Controlled Trials (CENTRAL, the Cochrane Library), MEDLINE via PubMed, and EMBASE databases from January 2016 (end of search for our 2016 systematic review) to August 2019. The principal search text word terms and MESH headings used are as follows: diarrhea/diarrhoea, diarrh*, gastroenteritis, probiotic*, *Lactobacillus reuteri*, as well as terms describing populations of interest (for details, see Table S1). We did not apply any language restrictions to our search strategy. Additionally, we screened the reference lists from identified studies and systematic reviews that were previously published. We also searched The ClinicalTrials.gov and ClinicalTrialsRegister.eu websites to identify potentially relevant unpublished RCTs. We did not consider for inclusion letters to the editor, abstracts, and proceedings from scientific meetings.

### 2.3. Collection of Data and Analysis 

Using a standardized form, the reviewers undertook the literature search, extraction of data, and quality assessment. The data extracted by one reviewer (BPG) included baseline characteristics, eligibility criteria, experimental and control treatments, study setting, dose of the intervention, and funding. The second reviewer (HS) evaluated all identified studies. In one case, we contacted the corresponding author [9] and obtained additional data.

### 2.4. Assessment of Risk of Bias in Included Studies 

The reviewers applied the Cochrane Collaboration’s tool for assessing the risk of bias of the included RCTs. The items that we assessed included methods of randomization (selection bias), allocation concealment (selection bias), blinding of participants and personnel (performance bias), blinding of outcome assessment (detection bias), and incomplete outcome data (attrition bias). Additionally, selective reporting and other types of bias were taken into account. If the evaluation was not feasible due to missing information, we rated the respective item as of unclear risk of bias.

### 2.5. Dealing with Missing Data 

We performed pooled data analysis with the use of available data for every participant (available case analysis), rather than intention-to-treat analysis with imputation.

### 2.6. Assessment of Heterogeneity and of Reporting Biases

Heterogeneity was quantified by *X*^2^ and *I*^2^, which can be interpreted as the percentage of the total variation between studies that is attributable to heterogeneity rather than to chance. No observed heterogeneity is indicated by a value of 0%, whereas larger values show increasing heterogeneity.

We planned to assess the publication bias using the funnel plot proposed by Egger et al. [15]. However, given the small number of studies (<10) included in the analyses, this was not formally assessed.

### 2.7. Data Synthesis (Statistical Methods) 

We analyzed the data with the use of Review Manager (RevMan [Computer program] Version 5.3 Copenhagen: The Nordic Cochrane Centre, The Cochrane Collaboration, 2014). The dichotomous outcomes, individual studies results, and pooled statistics were reported as the risk ratio (RR) between the experimental and control groups with 95% confidence intervals (95% CI). Mean difference (MD) between the treatment and control groups with 95% CI was reported for all continuous outcomes. The random-effects model was applied to all analyses.

For the primary outcomes, we planned to perform subgroup analyses based on factors potentially influencing the magnitude of the treatment response such as the dose of *L. reuteri* DSM 17938; setting (studies carried out in geographical Europe vs non-European countries); population (outpatient vs. inpatient); etiology of diarrhea; and vaccination against rotavirus status. However, there were not enough studies to perform these analyses.

In case of statistically significant heterogeneity in the primary outcome across studies, we performed subgroup analyses to determine the impacts of allocation concealment (adequate vs. inadequate or unclear) and blinding (open trial vs. double-blind trials). We also planned to evaluate whether the obtained results were affected by restricting these analyses to trials at low risk of bias only (defined as those with adequate randomization, allocation concealment, and blinding, at least 90% follow-up, and intention-to-treat analysis).

### 2.8. Certainty of Evidence 

We used the GRADE methodology and GRADE Profiler software, GRADEpro GDT: GRADEpro Guideline Development Tool [Software]. McMaster University, 2015 (developed by Evidence Prime, Inc.). Available from gradepro.org to assess the certainty of the evidence for outcomes that were reported in the included studies. The quality of evidence involves the within-study risk of bias (methodological quality), the directness of evidence, heterogeneity, effect estimates precision, and publication bias risk. Study quality refers to study methods and execution, such as the adequacy of allocation concealment, blinding, and follow-up. Consistency refers to the similarity of estimates of effect across studies. Directness refers to the extent to which the people, the interventions, and outcome measures are similar to those of interest. The GRADE system offers four categories of the quality of the evidence (high; moderate; low; and very low) [16].

## 3. Results

For a flow diagram documenting the identification process for eligible trials, see Figure S1. Detailed characteristics of the included RCTs are presented in Table 1. We excluded one trial due to reported co-intervention and comparison with a different probiotic [17]. Four RCTs that randomized 347 participants (172 in the experimental group and 175 in the control group) who ranged in age from 3 months to 5 years were identified [7,8,9,12]. The sample size in all included trials ranged from 60 to 127 participants. Included trials were carried out in countries such as Italy (1 RCT), Poland (1 RCT), and Turkey (2 RCTs). Three trials were carried out in inpatients and 1, in outpatients. The daily doses of *L. reuteri* DSM 17938 ranged from 1 × 10^8^ CFU (5 days) to 2 × 10^8^ CFU (5 days) to 4 × 10^8^ CFU (7 days). The comparator treatment was placebo in 2 trials and no intervention in 2 trials. In all studies, *L. reuteri* DSM 17938 was used in addition to rehydration therapy consisting of an oral rehydration solution and/or intravenous rehydration.

### 3.1. Risk of Bias in Included Studies 

High risk of bias was observed for at least one of the domains in the majority of the studies (see Figure S2).

### 3.2. Main Effects

*Stool volume.* None of the included trials assessed the effect of *L. reuteri* DSM 17938 on stool volume.

**Figure 1:**
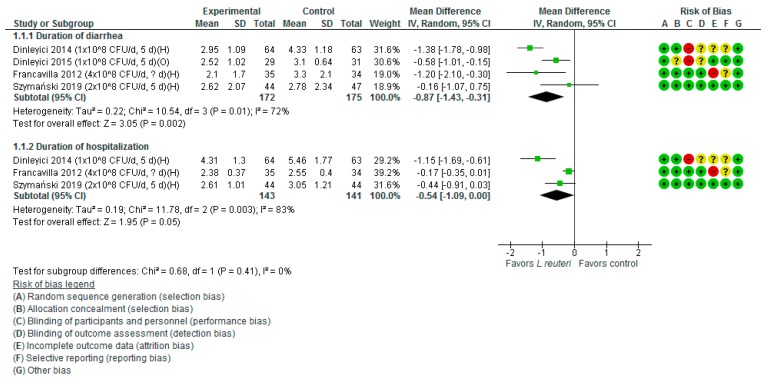
*L. reuteri* DSM 17938 versus control. Duration of diarrhea and hospitalization (days).

*Duration of diarrhea.* A meta-analysis of four trials (347 participants) showed a reduction in the duration of diarrhea for those treated with *L. reuteri* DSM 17938 compared with placebo or no treatment (MD −0.87 days, 95% CI −1.43 to −0.31; high heterogeneity, *I*^2^ = 72%) (Figure 1).

*Duration of hospitalization.* Compared with the placebo or no intervention groups, the use of *L. reuteri* DSM 17938 significantly reduced the duration of hospitalization; however, the difference was of a borderline statistical significance (three RCTs, *n* = 284, MD −0.54 days, 95% CI −1.09 to 0.0; high heterogeneity, *I*^2^ = 83%) (Figure 1).

**Figure 2:**
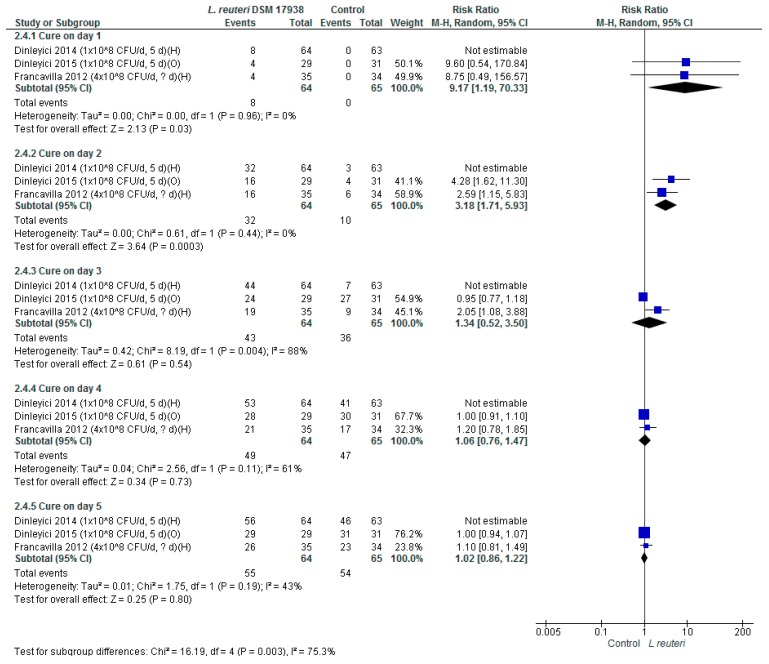
*L. reuteri* DSM 17938 versus control. Cure on any given day.

*Cure on any given day.* Based on the findings from three RCTs (*n* = 256), the use of *L. reuteri* DSM 17938, compared with placebo or no intervention, significantly increased the cure rate on both day 1 (RR 11.26, 95% CI 2.15 to 58.84) and day 2 (RR 4.54, 95% CI 2.02 to 10.18). However, no difference between the groups on days 3, 4, and 5 was observed (Figure 2).

**Figure 3:**
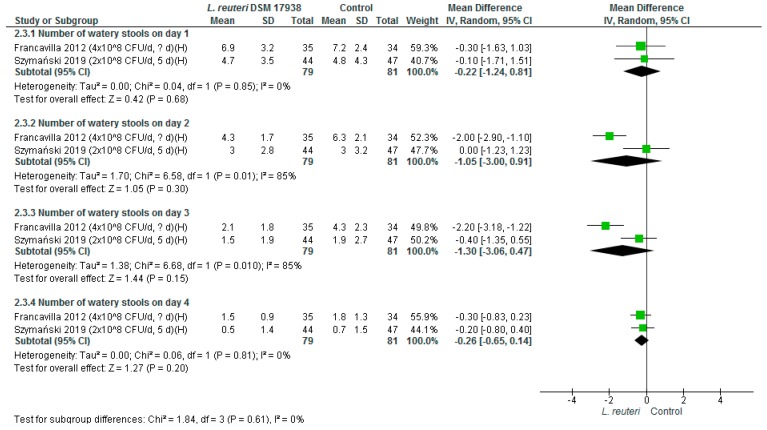
*L. reuteri* DSM 17938 versus control. Number of watery stools.

*Number of watery stools.* Based on the findings from two RCTs (*n* = 160), no significant differences were found between groups in the number of watery stools on days 1, 2, 3, and 4 (Figure 3).

*Adverse events.*

In all four studies, no adverse effects were observed, or their rates were similar in the experimental and control groups.

### 3.3. Subgroup and Sensitivity Analyses

Subgroup analysis based on allocation concealment and blinding did not substantially affect *I*^2^ (72% vs. 61%) or the effect size with respect to the duration of diarrhea (MD −0.68 days, 95% CI −1.70 to 0.34 in the subgroup with adequate allocation concealment and blinding). However, it did explain the observed heterogeneity between the studies with respect to the duration of hospitalization (*I*^2^ = 11%), showing an even smaller pooled effect size for studies with adequate blinding and allocation concealment (MD −0.22 days, 95% CI −0.42 to −0.02). Sensitivity analysis restricted to studies with low risk of bias (defined as above) resulted in the exclusion of three out of four studies. Therefore, no meta-analysis was performed. 

### 3.4. Certainty of Evidence

The GRADE assessment is presented in Table 2. Using the GRADE system, the overall certainty of evidence for all assessed outcomes was rated as low to very low. 

## 4. Discussion

### 4.1. Principle Findings

This updated meta-analysis of RCTs confirms the results of our previous analysis [6]. That is, in children with AGE, the addition of *L. reuteri* DSM 17938 to standard rehydration therapy compared with placebo or no intervention reduced the duration of diarrhea by approximately 21 h (very low certainty of the evidence). It also reduced hospitalization duration in inpatients by approximately 13 h (very low certainty), although the pooled effect size was smaller (5.3 h) when only studies with adequate blinding and allocation concealment were analyzed. The addition of *L. reuteri* DSM 17938 to standard rehydration therapy also increased the chance for cure on the first 2 days of treatment only; however, it had no significant effect on the number of watery stools. Adverse events were not observed, or rates were similar in both study groups.

### 4.2. Strengths and Limitations 

This review focuses on a single probiotic only; thus, it answers a clinically relevant question. We specifically assessed the effectiveness of *L. reuteri* DSM 17938, but not its original strain, *L. reuteri* ATCC 55730, which is known to carry potentially transferable resistance traits for tetracycline and lincomycin [18]. The use of the GRADE profile to rate the certainty of evidence makes our analysis useful for decision-making and guideline development. Still, the review has several important limitations. First, only a small number of studies were available. Second, unclear or high risk of bias in some of the included trials poses a question as to the reliability of the provided findings. Based on GRADE methodology, the overall certainty of evidence for all assessed outcomes was rated as low to very low. Third, due to the small number of trials (*N* = 4) available, trials with mixed (unclear, high, or low) levels of bias on some domains were combined in the analysis. Sensitivity analysis restricted to studies with low risk of bias resulted in the exclusion of three of the four studies, thus, no meta-analysis could be performed. Fourth, the significant heterogeneity between the studies can only in part be explained by methodological differences in study design. Different clinical factors likely contribute to this observed heterogeneity as well; however, given the small number of studies, meta-analyses in subgroups were not performed. The etiology of AGE, assessed in only two of the included trials, was mainly viral (rotavirus as a leading pathogen) or unknown. However, previous studies on different probiotics suggest that an enteropathogen-specific effect of probiotics seems unlikely [10,11]. The studies included in this review were conducted either in European countries or the transcontinental country of Turkey. Thus, our findings may require confirmation in other geographical regions of distinct populations. Another limitation is that the definitions of diarrhea and the duration of diarrhea differed. Some outcomes, such as the presence of diarrhea on any specific day of the intervention or the etiology of diarrhea, were evaluated in only a subset of trials with a limited number of participants; thereby, the findings, might be (non) significant by chance alone. Finally, due to the lack of data, it was not possible to definitively assess the effects of *L. reuteri* DSM 17938 on stool volume. 

### 4.3. Agreement and Disagreement with Other Studies or Reviews 

A number of systematic reviews have evaluated the efficacy of probiotics for the management of AGE in children [19,20,21,22,23,24,25,26]. However, only a few strains (single or in combination) were evaluated in two or more RCTs. A 2019 systematic review focused on *L. rhamnosus* GG [27]. Overall, 18 RCTs involving more than 4200 participants were included. Compared with placebo or no treatment, *L. rhamnosus* GG use was associated with a reduced duration of diarrhea by 0.85 days (95% CI −1.15 to −0.56) when all trials reporting this outcome (15 RCTs; 3820 participants) were evaluated. However, post hoc analysis of four RCTs (2280 participants) considered to be at low risk of bias with regard to adequate randomization, allocation concealment, blinding, and follow-up found that compared with controls, *L. rhamnosus* GG had no effect on the duration of diarrhea (MD −0.77 days, 95% CI −2.01 to 0.47). A recent re-analysis of data from these four higher-quality trials (no or only one domain of unclear risk of bias) that included an additional RCT (5 RCTs; 2429 participants) similarly found that, compared with controls, *L. rhamnosus* GG had no effect on the duration of diarrhea (MD −0.68 days, 95% CI −1.81 to 0.44) [28].

Three systematic reviews [29,30,31] focused on *Saccharomyces boulardii.* Although these reviews differed with respect to inclusion/exclusion criteria, they consistently reported that, compared with the placebo or no intervention groups, the use of *S boulardii* significantly reduced the duration of diarrhea. However, the overall quality of the trials was low. Other probiotics studied in two or more trials include *Bacillus clausii* strains O/C, SIN, N/R, and T [32]; *L. helveticus* R0052 and *L. rhamnosus* R0011 [11,33,34,35]; *L. rhamnosus* 19070–2 and *L. reuteri* DSM 12246 [36,37]; and *Bacillus mesentericus* TO-A, *Enterococcus faecalis* T-110, and *Clostridium butyricum* TO-A [38,39]. Both positive and negative effects were found in these trials, further confirming our assertion that each probiotic needs to be evaluated separately.

To the best of our knowledge, there are no reported data that *L. reuteri* DSM 17938 causes any serious adverse events, including when used in preterm infants [40]. Moreover, *L. reuteri* DSM 17938 has been granted generally regarded as safe (GRAS) status in the US and qualified presumption of safety (QPS) status in the EU.

## 5. Conclusions

Current evidence shows that, overall, *L. reuteri* DSM 17938 reduced the duration of diarrhea and hospitalization of inpatients. These findings of limited clinical significance due to small effect sizes should be further viewed with caution in the context of the heterogeneity and methodological limitations of the included trials. The findings of this review may inform guideline development groups about the efficacy of *L. reuteri* DSM 17938 for treating children with AGE.

## Figures and Tables

**Table 1:** Characteristics of the included studies.

	Study ID; Country	Participants (Exp/Cont)	Intervention— *L. reuteri* DSM 17938 (Dose; Duration)	Comparison	Inclusion Criteria	Sample Size Calculation	Funding
Treatment of Acute Gastroenteritis							
1.	Dinleyici 2014; Turkey	64/63	1 × 10^8^ CFU; 5 days	No intervention	3–60 months; acute diarrhea (no definition) lasting 12–72 h; mild to moderate dehydration; hospitalization	Yes	The study was not funded (except for the free medication)
2.	Dinleyici 2015; Turkey	29/31	1 × 10^8^ CFU; 5 days	No intervention	3–60 months; acute diarrhea (≥3 loose or watery stools per 24 h) lasting 12–72 h; outpatients	Yes	Not described
3.	Francavilla 2012; Italy	35/34	4 × 10^8^ CFU; 7 days	Placebo (mixture of sunflower oil and medium-chain triglyceride oil)	Age 6–36 months; acute diarrhea (≥2 or watery stools per 24 h) of no more than 7 days duration; mild to moderate dehydration; hospitalization	Yes	Not described
4.	Szymański 2019; Poland	44/47	2 × 10^8^ CFU; 5 days	Placebo	Age < 5 years; acute gastroenteritis (a change in stool consistency to a loose or liquid form [according to the BSF scale, or, in the case of infants, the ASF scale] and/or an increase in the frequency of evacuations, typically ≥ 3 in 24 h), lasting no longer than 5 days.	Yes	BioGaia provided the study products. The study received no external funding.

ASF: Amsterdam Stool Form; BSF: Bristol Stool Form; CFU: colony-forming units.

**Table 2:** GRADE assessment for the primary outcomes.

Patient or Population: Acute Gastroenteritis in Children Setting: Intervention: *Lactobacillus reuteri* DSM 17938 Comparison: Placebo/No Treatment						
Outcomes	Anticipated Absolute Effects * (95% CI)		Relative Effect (95% CI)	№ of Participants (Studies)	Certainty of the Evidence (GRADE)	Comments
	Risk With Placebo/No Treatment	Risk With Lactobacillus Reuteri DSM 17938				
*Lactobacillus reuteri* DSM 17938 vs control. Duration of diarrhea (days)	The mean lactobacillus reuteri DSM 17938 vs control. Duration of diarrhea (days) ranged from **0.64 to 2.34** days	MD **0.87 days lower** (1.43 lower to 0.31 lower)	-	347 (4 RCTs)	⨁◯◯◯ VERY LOW ^a,b,c,d^	
*Lactobacillus reuteri* DSM 17938 vs. control. Duration of hospitalization	The mean lactobacillus reuteri DSM 17938 vs. control. Duration of hospitalization ranged from **2.55 to 5.46** days	MD **0.54 days lower** (1.09 lower to 0)	-	284 (3 RCTs)	⨁◯◯◯ VERY LOW ^d,e,f,g^	
**GRADE Working Group grades of evidence** **High certainty:** We are very confident that the true effect lies close to that of the estimate of the effect **Moderate certainty:** We are moderately confident in the effect estimate: The true effect is likely to be close to the estimate of the effect, but there is a possibility that it is substantially different **Low certainty:** Our confidence in the effect estimate is limited: The true effect may be substantially different from the estimate of the effect **Very low certainty:** We have very little confidence in the effect estimate: The true effect is likely to be substantially different from the estimate of effect						

^a^. Two out of four studies open label. ^b^. Large heterogeneity *I*^2^ = 74% (*p* < 0.05). ^c^. Differences in dosage across studies; two out of four included studies on a non-European population. ^d^. The recommendation would be altered if the lower versus the upper boundary of the CI represented the true underlying effect (based on the assumption that 1-day reduction is clinically important). ^e^. Population differences (European and non-European). ^f^. No blinding in one out of three studies. ^g^. Test for heterogeneity *p* < 0.05; *I*^2^ = 83%. * The risk in the intervention group (and its 95% confidence interval) is based on the assumed risk in the comparison group and the relative effect of the intervention (and its 95% CI). CI: Confidence interval; MD: Mean difference; RR: Risk ratio.

## Supplementary Materials

Additional Supporting Information may be found in https://www.mdpi.com/2072-6643/11/11/2762/s1: Figure S1: The identification process for eligible trials (since January 2016, i.e., the date of the last search), Figure S2: Risk of bias in the included studies, Table S1: Example of search strategy for EMBASE.
