# Supplementary material for: Systematic Review with Meta-Analysis: Lactobacillus reuteri DSM 17938 for Treating Acute Gastroenteritis in Children. An Update
Source: Nutrients. 2019 Nov 14;11(11):2762. doi: 10.3390/nu11112762 (PMC6893691; doi:10.3390/nu11112762)

SUPPLEMENTARY MATERIALS

Systematic review with meta-analysis: *Lactobacillus reuteri* DSM 17938 for treating acute gastroenteritis in children. An update

Bernadeta Patro-Gołąb[1], Hania Szajewska[1].
[1]Department of Paediatrics, The Medical University of Warsaw

Corresponding author:
Hania Szajewska, MD
The Medical University of Warsaw
Department of Paediatrics
02-091 Warsaw, Żwirki i Wigury 63A

Table S1. Example of search strategy for EMBASE.

(((('lactobacillus' OR 'lactobacillus'/exp OR lactobacillus) AND reuteri OR l.) AND reuteri OR dsm) AND 17938 OR 'probiotic' OR 'probiotic'/exp OR probiotic OR 'probiotics' OR 'probiotics'/exp OR probiotics) AND ('pediatric' OR 'pediatric'/exp OR pediatric OR 'paediatric' OR 'paediatric'/exp OR paediatric OR 'child' OR 'child'/exp OR child OR 'children' OR 'children'/exp OR children OR 'infants' OR 'infants'/exp OR infants OR 'toddlers' OR 'toddlers'/exp OR toddlers OR 'adolescents' OR 'adolescents'/exp OR adolescents OR newborns) AND ('diarrhea' OR 'diarrhea'/exp OR diarrhea OR 'diarrhoea' OR 'diarrhoea'/exp OR diarrhoea OR diarrh* OR 'gastroenteritis' OR 'gastroenteritis'/exp OR gastroenteritis) AND [2016-2019]/py AND [embase]/lim

Figure S1. The identification process for eligible trials (since January 2016, i.e., the date of the last search).

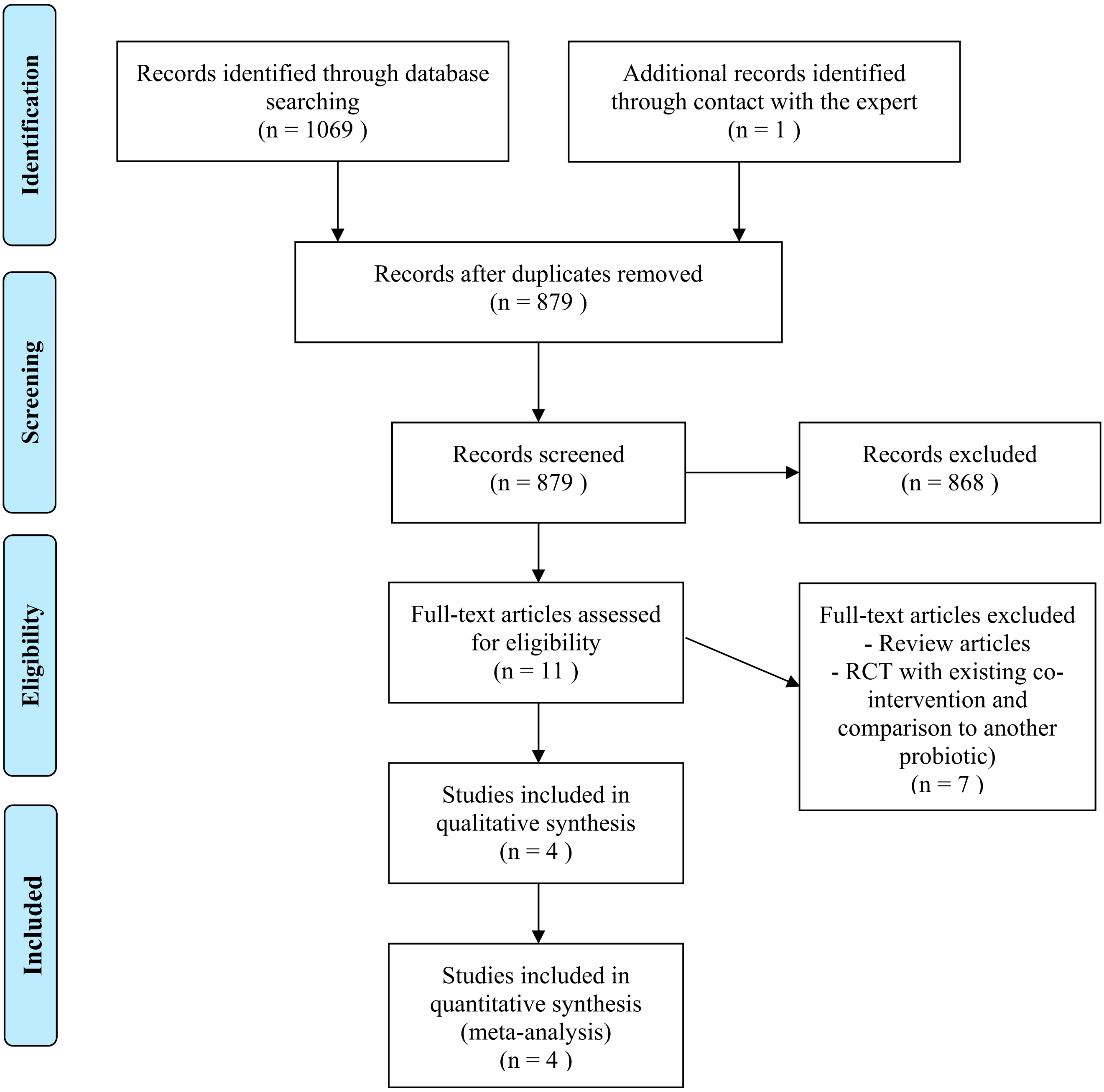

Figure S2. Risk of bias in the included studies.

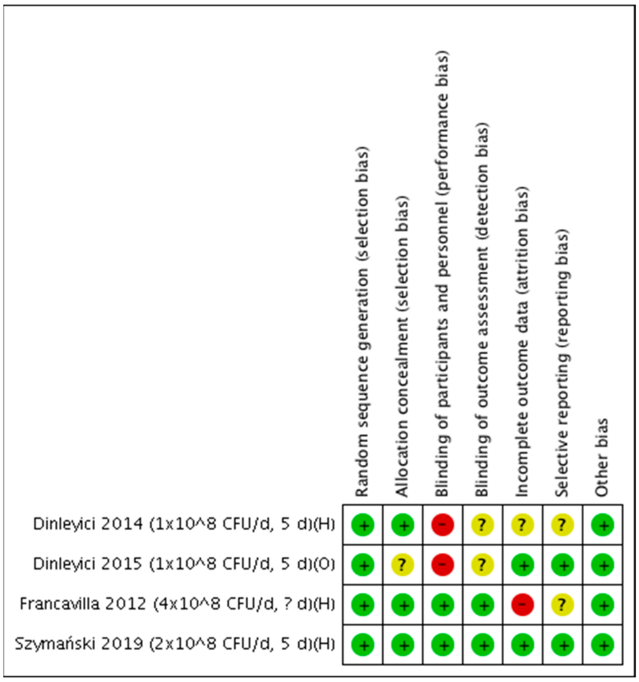

Supplement: Supplementary file 1 [file nutrients-11-02762-s001.pdf]
